# Comparison of left atrial and left atrial appendage mechanics in the risk stratification of stroke in patients with atrial fibrillation

**DOI:** 10.1186/s12947-020-00232-z

**Published:** 2021-01-09

**Authors:** Yankai Mao, Chan Yu, Yuan Yang, Mingming Ma, Yunhe Wang, Ruhong Jiang, Ran Chen, Bowen Zhao, Chenyang Jiang

**Affiliations:** 1 Department of Diagnostic Ultrasound & Echocardiography, Sir Run Run Shaw Hospital, Zhejiang University School of Medicine, #3 East Qingchun Road, Hangzhou, China; 2 Department of Cardiology, Sir Run Run Shaw Hospital, Zhejiang University School of Medicine, 3 East Qingchun Road, Hangzhou, People’s Republic of China

**Keywords:** Left atrium, Left atrial appendage, Mechanical dispersion, Speckle-tracking echocardiography, Stroke, Atrial fibrillation

## Abstract

**Background:**

Left atrial (LA) and left atrial appendage (LAA) dysfunction has been demonstrated to contribute to atrial fibrillation (AF)-related stroke. However, usefulness of LA and LAA mechanics has not been fully compared. We sought to investigate the association of LA and LAA mechanics with stroke and to compare their diagnostic values in the risk stratification of stroke in patients with nonvalvular AF.

**Methods:**

A total of 208 consecutive patients with AF (63.58 ± 10.37 years, 63.9% male,57.7% persistent AF) who underwent echocardiography before catheter ablation were prospectively enrolled. Speckle-tracking was used to measure LA and LAA global longitudinal strain (GLS). LA and LAA mechanical dispersions (MD) were defined as the standard deviation (SD) of time to peak positive strain corrected by the R-R interval.

**Results:**

Patients with prior stroke/ transient ischemic attack (TIA) (*n* = 31) had significantly higher LA and LAA MD than those without (*n* = 177) (11.56 ± 4.38% vs. 8.43 ± 3.44%, 15.15 ± 5.46% vs. 10.94 ± 4.40%, both *P* < 0.01). In multivariable analysis, LA and LAA MD were independently associated with stroke/TIA (odds ratio, 1.18–1.29, 1.19–1.22, respectively, both *P* < 0.01), providing incremental values over clinical and standard echocardiographic parameters. In a subgroup analysis, LA MD was more useful than LAA MD in patients with normal LA volumes, while LAA MD was superior to LA MD in patients with LA enlargement.

**Conclusions:**

Higher LA and LAA mechanical dispersion are independently associated with stroke/TIA in AF patients and had incremental values over clinical and conventional echocardiographic parameters. What’s more, priorities of dispersion assessment are different depending on patients’ LA size.

**Supplementary Information:**

The online version contains supplementary material available at 10.1186/s12947-020-00232-z.

## Introduction

Non-valvular atrial fibrillation (AF) is independently associated with 5-fold increased risk of ischaemic stroke [1]. As the most devastating complication of AF, cardioembolic strokes related to AF confer increased risk of mortality and worse outcomes than non-AF strokes [2]. Currently, CHA2DS2-VASc scoring system is the widely used for estimating stroke risk in AF patient [3]. However, its direct mechanistic link with AF-related stroke has yet to be identified and its accuracy to predict ischemic stroke is modest, especially in those with a score of < 2 [4].

AF is associated with left atrium (LA) and left atrial appendage (LAA) remodeling, which includes cavity dilation, myocardial fibrosis and subsequent dysfunction [5–7]. Although irregular contraction and subsequent intracardiac thrombosis has long been considered a direct mechanism for AF-related strokes [8], recent studies suggest that structural and functional abnormalities of LA/LAA may also contribute to stroke/ transient ischemic attack (TIA) [9–12]. LA enlargement, presence of spontaneous echo contrast (SEC) and thrombus in LA/LAA and reduced LAA emptying velocity (LAAEV) are well-established markers of stroke risk [9, 13, 14], but stroke often occurs in patients without LA enlargement or reduced LAAEV. Speckle-tracking echocardiography (STE) provides an accurate assessment of subclinical myocardial dysfunction [15], and impaired LA and LAA global longitudinal strain (GLS) are related to stroke or embolism [12, 16–18]. Myocardial strain analysis may also measure the timing of contraction, and recent studies have demonstrated an independent association between LA and LAA mechanical dispersion (MD) and the presence of LAA thrombi or sludge [19] or stroke in AF patients [20, 21]. However, although LAA MD improved the risk stratification of embolism [21], its assessment is complicated and time-consuming. In contrast, LA MD is easier to obtain and has been validated in different clinical settings to estimate subclinical LA dysfunction and can be potentially useful for predicting a variety of cardiovascular diseases, including stroke/TIA [19, 20, 22, 23]. However, usefulness of both LA and LAA mechanics has not been fully compared for identification of stroke. This study aims to determine the associations of LA and LAA MD with stroke/TIA and to compare their incremental values in the risk stratification for stroke in patients with nonvalvular AF.

## Methods and materials

### Study population

We performed a cross-sectional study using 249 prospectively enrolled patients with non-valvular AF referred to Sir Run Run Shaw Hospital for catheter ablation between April 2019 and March 2020. All patients underwent preprocedural transthoracic echocardiography (TTE) and transesophageal echocardiography (TEE) with subsequent STE. We excluded 29 patients either with congenital heart disease (*n* = 4), history of any cardiac surgery and/or cardiac device implantation(*n* = 7), cardiac mass(*n* = 1), cardiomyopathies(*n* = 6) and apparent carotid atherosclerosis(*n* = 4), and inadequate image quality hampering strain analysis(*n* = 7). We also excluded patients who were in sinus rhythm at the time of echocardiography (*n* = 12). Patients were carefully assessed for the history of stroke or TIA. Ischemic stroke was defined by a history of hospital admission, a focal neurologic deficit of sudden onset and positive imaging findings [16]. TIA refers to neurologic signs and symptoms resolved within 24 h with negative imaging finding. The final population of 208 patients were classified into stroke group (with a history of stroke/ TIA before admission, *n* = 31) and the control group (*n* = 177). The patients were classified as having either paroxysmal or persistent AF based on the guidelines [24].Clinical parameters including demographic variables, medical history and medication history were recorded. The thromboembolic risk was assessed using the CHA_2_DS_2_-VASc scores before stroke/TIA. The study protocol was approved by the local institutional review board and was conducted in accordance with the Declaration of Helsinki and its later amendments. All participants provided their written, informed consent.

### Standard echocardiography

All patients routinely underwent TTE and TEE using a Vivid E95 echocardiograph(GE Vingmed Ultrasound AS, Horten Norway) equipped with a M5Sc (1.4–4.6 MHz) probe and a multiplane 6VT (3.0–8.0 MHz) transducer. Standard echocardiographic parameters were measured according to current recommendations [25], including left ventricular (LV) and LA volumes, LV ejection fraction (LVEF) and LA antero-posterior diameter (LAAPd). Lidocaine hydrochloride spray was used for local anaesthesia before TEE studies. A comprehensive visual assessment of LAA was performed at the mid-esophageal position by sweeping from 0°-180°. The LA and LAA were examined for the presence of dense SEC or thrombus. The dense SEC was defined as very slow swirling smoke-like echoes detectable within the LA/LAA throughout the cardiac cycle. A thrombus was defined as a mobile, irregularly shaped, echo-dense mass that was clearly distinct from LA endocardium and pectinate muscles. LA volume was measured using the area-length method from the apical four and two chamber views. The LAA volume was determined using the same method from two orthogonal views typically at 45° and 135°.The LA and LAA emptying fraction (LAEF and LAAEF) was calculated as [maximum volume (V_max_)-minimal volume (V_min_)]/V_max_*100. LAA emptying velocity (EV) and filling velocity (FV) was also recorded. All linear and volumetric variables were subsequently indexed to body surface area (BSA).

### Speckle-tracking echocardiography

Five consecutive cardiac cycles were stored in cine-loop format for strain analysis, which was performed with vendor-dependent software (EchoPAC PC version 203, GE Vingmed Ultrasound AS, Horten Norway). We used images acquired with a frame rate of 60–80 frames/sec during breath hold. The LA endocardial border was manually traced in both four-chamber and two-chamber views. The LAA endocardium was manually traced from mid-esophageal TEE views obtained at 0°,45°,90° and 135°.The endo-and epicardial border tracing were adjusted thereafter so that the region of interest covered full thickness of LA or LAA wall. The software divided the LA or LAA wall into 6 segments in each view and generated strain curves for each segment. Any segments that failed to track were rejected and excluded from analysis. LA or LAA GLS was obtained by averaging peak positive strain values in all segments if they were tracked adequately. LA or LAA MD was defined as the standard deviation (SD) of the time to peak positive strain of each segment and expressed as a percentage of the R-R’ interval. Time-to-peaks in opposite phase to the expected direction of strains were not included in the final computation. Higher values of LA or LAA MD indicate a greater degree of LA or LAA dyssynchrony. The reference frame of zero strain was set at LV end-diastole (R-R gating) [26]. To resolve the problem of beat-to-beat variation in STE measurements we used the index-beat method [19, 27]. Each LA or LAA measurement was estimated using the ratio of the preceding to prepreceding R-R′ interval. All echocardiographic analysis was performed by one investigator experienced with strain imaging and blinded to the patients’ information. Of the total of 4992 LAA segments and 2496 LA segments analyzed in 208 patients, STE analysis was feasible in 6968 (93.1%) segments.

### Statistical analysis

IBM SPSS package 25.0 (SPSS, Inc., Chicago, IL, USA) was used to perform the statistical analyses. Continuous data were presented as mean ± SD. Categorical variables were expressed as numbers and percentages. Comparisons between groups were performed by using independent Student’s t-test, the Mann-Whitney U test, Chi-square test or Fisher’s exact test where appropriate. Univariate and multivariate binary logistic regression analysis was used to assess the associations between clinical or echocardiographic parameters and prior stroke/TIA. The independence and robustness of LA MD and LAA MD were examined using several models. Risks were expressed as odds ratio (OR) with 95% confidence interval (CI). The incremental values of LA MD and LAA MD over clinical characteristics and conventional echocardiographic parameters were assessed in the overall group and in subgroups (patients with normal and abnormal LA volumes). Covariate selection for model entry was based on our own hypothesis and previous findings. The incremental values of LA and LAA MD were determined by comparing the improvement in global χ^2^ value for each model. We generated receiver operating characteristic (ROC) curve to determine the ability of different variables in identifying stroke/TIA, and the area under the curve (AUC) was compared. The cut-off value was obtained using the criterion corresponding to the highest Youden index.

Inter- and intra-observer variability for LA/LAA GLS and MD were studied in 15 randomly selected patients by two independent investigators on two different occasions. Reproducibility was expressed as intra-class correlation coefficient (ICC). Statistical significance was defined as *P* < 0.05.

## Results

### Patient characteristics

Demographic, clinical, and echocardiographic data for the study population are presented in Table 1. A total of 208 patients (63.58 ± 10.37 years, 63.9% male, 57.7% persistent AF) were included in the final analysis. The stroke group were older, had higher CHA2DS2-VASc scores before stroke (2.29 ± 1.16 vs1.73 ± 1.26, *P* = 0.02), higher prevalence of LAA dense SEC or thrombi, and more frequently suffered from heart failure than the control group. In the stroke group, 11 patients (35.5%) were on anticoagulation before stroke/TIA, while 12 patients (38.7%) started anticoagulation after stroke/TIA, and the remaining 8 patients (25.8%) did not use anticoagulants. Other clinical characteristics did not show significant differences between two groups.

**Table 1:** Clinical and echocardiographic characteristics of the study population

	Overall (*N* = 208)	Stroke/TIA (*N* = 31)	Control (*N* = 177)	*P* value
**Clinical characteristics**				
Gender,male	133 (63.9)	20 (64.5)	113 (63.8)	0.94
Age,years	63.58 ± 10.37	68.32 ± 7.57	62.75 ± 10.59	< 0.01
Body mass index,kg/m^2^	24.37 ± 3.20	24.72 ± 3.07	24.31 ± 3.22	0.52
Heart failure	33 (15.9)	10 (32.3)	23 (13.0)	< 0.01
Coronary artery disease	37 (17.8)	7 (22.6)	30 (16.9)	0.45
Hypertension	111 (53.4)	19 (61.3)	92 (52.0)	0.34
Diabetes	45 (21.6)	8 (25.8)	37 (20.9)	0.54
Hyperlipoproteinemia	58 (27.9)	5 (16.1)	53 (29.9)	0.11
Persistent AF	120 (57.7)	20 (64.5)	100 (56.5)	0.40
Anticoagulation	145 (69.7)	23 (74.2)	122 (68.9)	0.56
CHA_2_DS_2_-VASc score before stroke	1.82 ± 1.26	2.29 ± 1.16	1.73 ± 1.26	0.02
**Conventional echocardiographic parameters**				
iLVESV,mL/m2	24.18 ± 9.40	27.82 ± 14.40	23.56 ± 8.17	0.13
iLVEDV,mL/m2	66.84 ± 14.55	70.56 ± 17.95	66.21 ± 13.85	0.13
LVEF,%	64.26 ± 8.24	61.58 ± 8.73	64.72 ± 8.09	0.05
iLAAPd,mm/ m2	22.77 ± 3.82	23.48 ± 3.72	22.64 ± 3.83	0.26
iLAVmin,mL/m2	27.88 ± 15.48	34.86 ± 18.81	26.63 ± 14.53	< 0.01
iLAVmax,,mL/m2	41.67 ± 17.08	47.79 ± 22.96	40.57 ± 15.63	0.03
LAEF, %	35.2 ± 16.00	28.56 ± 13.69	36.39 ± 16.13	0.01
LAAEV,m/s	0.53 ± 0.24	0.45 ± 0.25	0.54 ± 0.23	0.09
LAAFV,m/s	0.53 ± 0.20	0.47 ± 0.23	0.54 ± 0.20	0.10
LAA dense SEC/thrombus	36 (18.5)	11 (40.7)	25 (14.9)	< 0.01
ilAAVmin,mL/m2	1.89 ± 1.58	2.06 ± 1.53	1.86 ± 1.59	0.53
iLAAVmax,mL/m2	4.24 ± 2.41	4.12 ± 2.58	4.26 ± 2.38	0.77
LAAEF,%	57.30 ± 19.83	51.86 ± 16.81	58.19 ± 20.19	0.13
**Strain echocardiographic parameters**				
LA GLS,%	18.08 ± 9.64	13.09 ± 7.01	19.99 ± 9.79	< 0.01
LA MD,%	8.91 ± 3.76	11.56 ± 4.38	8.43 ± 3.44	< 0.01
LAA GLS,%	12.15 ± 5.82	9.01 ± 3.12	12.66 ± 6.00	< 0.01
LAA MD,%	11.53 ± 4.78	15.15 ± 5.46	10.94 ± 4.40	< 0.01

Data are expressed as mean ± SD or N (%)

*AF* atrial fibrillation, *EDV* end-diastolic volume, ESV end-systolic volume, *GLS* global longitudinal strain, *i* indexed to body surface area, *LA* left atrium, *LAA* left atrial appendage, *LAA EF* LAA emptying fraction, *LAAEV* LAA emptying velocity, *LAAFV* LAA filling velocity, *LAAPd* LA anteroposterior diameter, *LAEF* LA emptying fraction, *LV* left ventricle, *LVEF* left ventricular ejection fraction, *MD* mechanical dispersion, *SEC* spontaneous echo contrast, *TIA* transient ischaemic attack, *Vmax* maximal volume, *Vmin* minimal volume

**Fig. 1:**
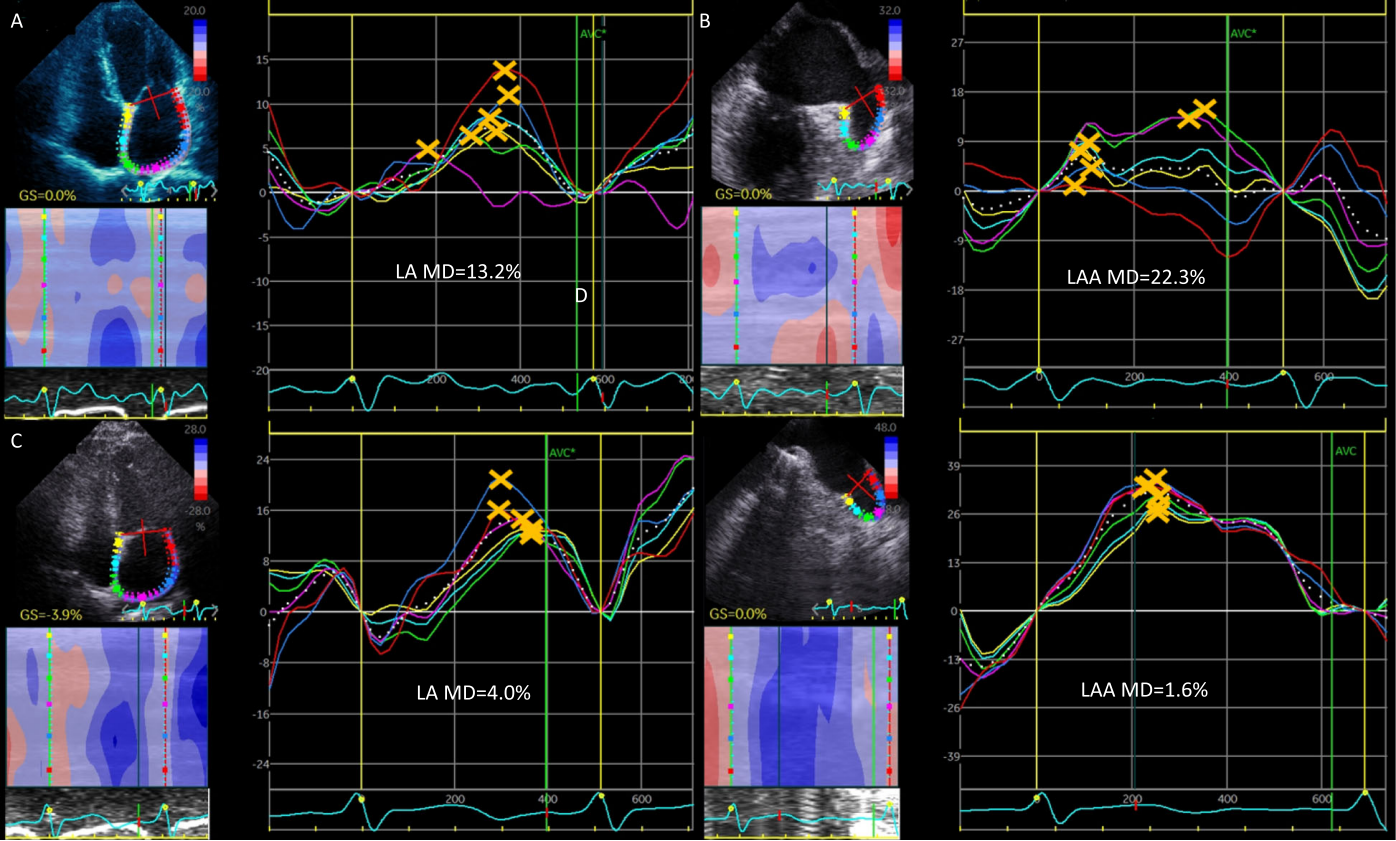
LA and LAA strain curves from speckle-tracking in patients with stroke (**a**, **b**) and without stroke (**c**, **d**). Yellow crosses indicate the positive peaks of each curve. LA and LAA MD was calculated as the SD of time to peak and expressed as a percentage of the R-R interval. The patients with stroke showed higher LA and LAA MD. LA, left atrium; LAA, left atrial appendage; MD, mechanical dispersion

Patients in the stroke group had higher indexed LA volumes (Vmax and Vmin), lower LAEF, depressed LA and LAA GLS, suggesting that these patients had impaired LA and LAA function compared with controls. Furthermore, the stroke group showed more pronounced LA MD (11.56 ± 4.38% vs 8.43 ± 3.44%, *P* < 0.01) and LAA MD (15.15 ± 5.46% vs 10.94 ± 4.40%, *P* < 0.01) than those of the control group. Figure 1 shows representative cases of LA and LAA strain curves and MD in patients with and without stroke.

### Factors associated with prior stroke/TIA

The univariate logistic regression analysis identified a variety of clinical, TTE and TEE parameters as significant contributors to prior stroke/TIA. Age, LA MD and LAA MD remained to be independent discriminators in each multivariate model with similar ORs (Age,1.06–1.08, *P* < 0.05; LA MD,1.18–1.29, *P* < 0.01; LAA MD, 1.19–1.22, *P* < 0.01) (Table 2).

**Table 2:** Univariable and multivariable logistic regression analyses of associations between clinical and echocardiographic covariates with stroke

Variables	Univariate analysis		Multivariate analysis					
	OR (95% CI)	***P*** value	Model 1		Model 2		Model 3	
			OR (95% CI)	***P*** value	OR (95% CI)	***P*** value	OR (95% CI)	***P*** value
Clinical parameters								
Age	1.07 (1.02–1.12)	< 0.01	1.06 (1.01–1.13)	0.03	1.07 (1.01–1.14)	0.03	1.08 (1.01–1.40)	0.02
Female	1.03 (0.46–2.29)	0.94	2.17 (0.79–6.02)	0.14	2.44 (0.85–7.03)	0.09		
Heart failure	3.19 (1.33–7.62)	0.01	1.85 (0.63–5.45)	0.27	1.56 (0.48–5.24)	0.46		
CHA2DS2-VASc score	1.41 (1.04–1.19)	0.03	1.04 (0.55–1.97)	0.91	1.14 (0.57–2.29)	0.71	1.07 (0.57–1.63)	0.71
**LA parameters**								
LAEF	0.97 (0.94–0.99)	0.01	1.00 (0.96–1.04)	0.99				
iLAVmax	1.02 (1.00–1.04)	0.03	0.99 (0.96–1.02)	0.53			0.99 (0.96–1.03)	0.81
LA GLS	0.91 (0.86–0.97)	< 0.01	0.99 (0.90–1.09)	0.88	0.99 (0.89–1.10)	0.77	0.99 (0.89–1.09)	0.78
LA MD	1.25 (1.22–1.39)	< 0.01	1.20 (1.07–1.36)	< 0.01	1.29 (1.11–1.49)	< 0.01	1.18 (1.03–1.34)	< 0.01
**LAA parameters**								
LAAEV	0.18 (0.02–1.33)	0.09			16.81 (1.28–220.9)	0.03	4.83 (0.45–52.15)	0.05
LAAEF	0.98 (0.97–1.00)	0.13			1.01 (0.98–1.05)	0.45		
LAA dense SEC/thrombus	3.93 (1.64–9.46)	< 0.01			4.39 (1.33–14.53)	0.02	3.63 (1.16–11.35)	0.03
LAA GLS	0.84 (0.75–0.94)	< 0.01	0.96 (0.85–1.09)	0.53	0.96 (0.84–1.10)	0.57	0.96 (0.84–1.09)	0.49
LAA MD	1.21 (1.09–1.32)	< 0.01	1.19 (1.07–1.33)	< 0.01	1.22 (1.08–1.38)	< 0.01	1.21 (1.08–1.36)	< 0.01

*AF* atrial fibrillation, *BMI* body mass index, *CI* Confidence intervals, *GLS* global longitudinal strain, *i* indexed to body surface area, *LA* left atrium, *LAA* left atrial appendage, *LAAEF* LAA emptying fraction, *LAAEV* LAA emptying velocity, *LAEF* LA emptying fraction, *MD* mechanical dispersion, *OR* odds ratio, *SEC* spontaneous echo contrast, *Vmax* maximal volume

The ROC curve analysis results are summarized in the Supplementary data Table 1. The AUC for most STE parameters were higher than clinical and standard echocardiographic variables, with LA MD and LAA MD having the highest diagnostic performance (AUC 0.724, 0.771, 95% CI 0.666–0.777, 0.714–0.822, respectively). Using a LA MD cut-off value of > 11.47% or LAA MD cut-off value of > 12.97%, patients with stroke/TIA were identified with a sensitivity of 51.61%, 85.19% and specificity of 82.94%, 66.87%, respectively.

### Incremental value of LA MD and LAA MD in risk stratification of stroke

**Fig. 2:**
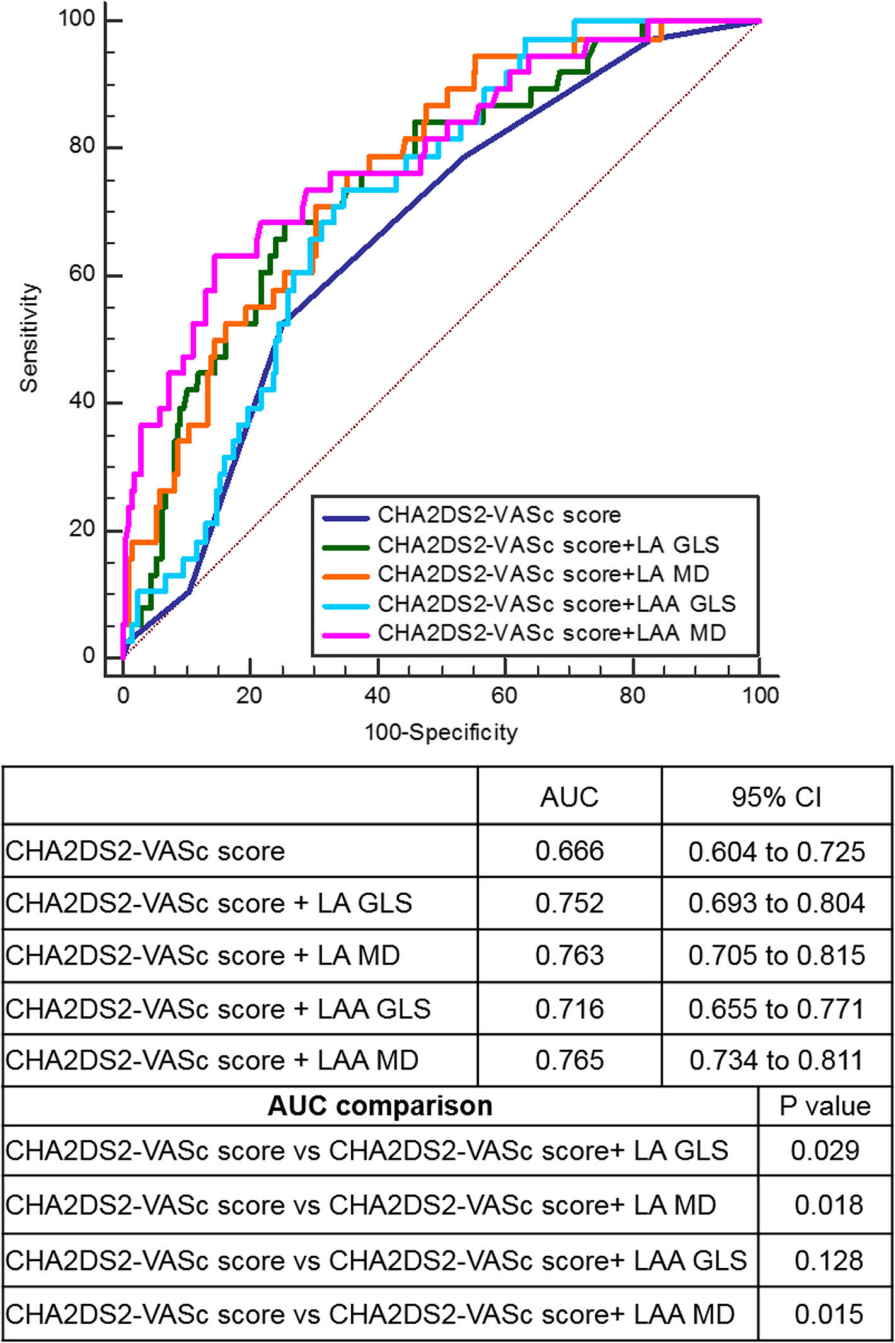
Results of receiver-operating characteristic curve analysis for identifying stroke in all patients. AUC, area under the curve; GLS, global longitudinal strain; LA, left atrium; LAA, left atrial appendage; MD, mechanical dispersion

**Fig. 3:**
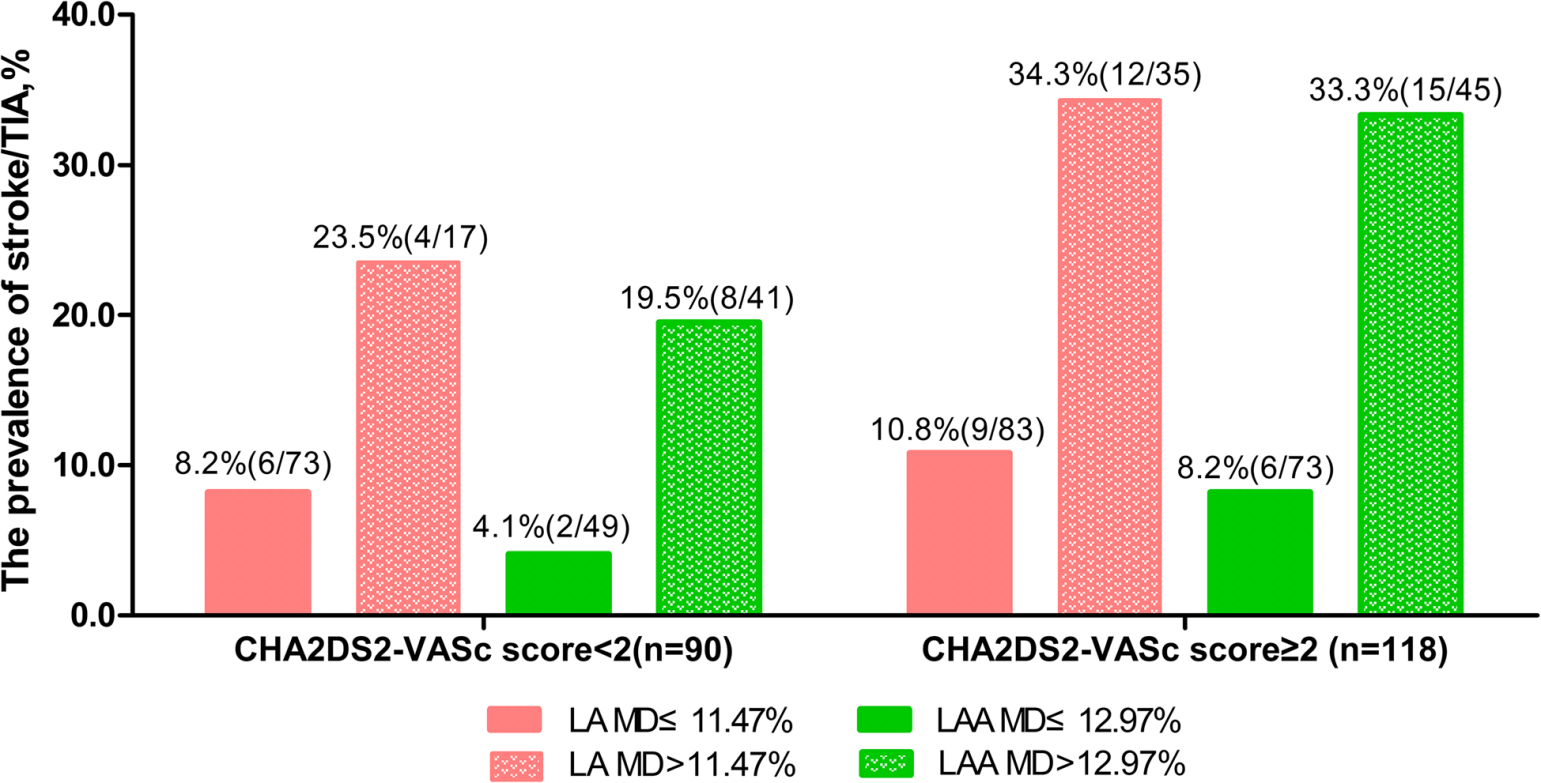
The prevalence of stroke according to LA or LAA MD and CHA2DS2-VASc score. LA, left atrium; LAA, left atrial appendage; MD, mechanical dispersion

**Fig. 4:**
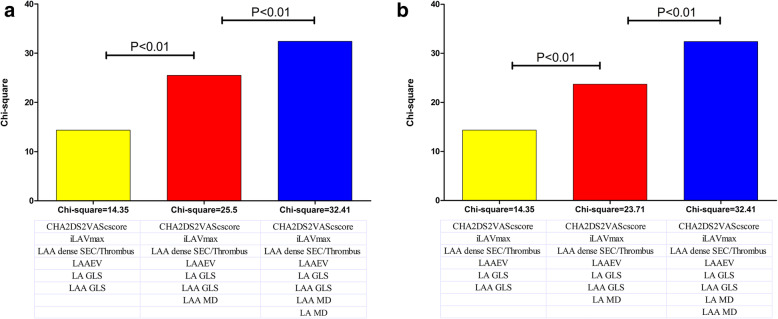
Incremental values of LA and LAA MD for stroke risk stratification. **a** The initial model based on clinical and conventional echocardiographic parameters as well as strains was significantly improved by the addition of LA MD and further improved by adding LAA MD. **b** The same initial model was also significantly improved by the addition of LAA MD and further improved by adding LA MD. AF, atrial fibrillation; GLS, global longitudinal strain; i, indexed to body surface area; LA, left atrium; LAA, left atrial appendage; LAAEV, LAA emptying velocity; MD, mechanical dispersion; SEC, spontaneous echo contrast; Vmax, maximal volume

ROC analysis showed that the AUCs of CHA2DS2-VASc score plus one of each strain (LA GLS, LAA GLS, LA MD, LAA MD) models were significantly higher than that of the CHA2DS2-VASc score alone (Fig. 2). Moreover, we summarized the prevalence of stroke/TIA according to different LA MD or LAA MD and CHA2DS2-VASc score in Fig. 3. In the patients with CHA2DS2-VASc score ≥ 2 (*n* = 118) or < 2 (*n* = 90), the prevalence of stroke/TIA was significantly higher in patients with LA MD > 11.47% (34.3% (12/35) vs 10.8% (9/83), *P* < 0.01; 23.5% (4/17) vs 8.2% (6/73), *P* < 0.01, respectively) or LAA MD > 12.97% (33.3% (15/45) vs 8.2% (6/73), *P* < 0.01; 19.5% (8/41) vs 4.1% (2/49), *P* < 0.01, respectively) than that of patients with LA MD ≤ 11.47% or LAA MD ≤ 12.97%. Finally, we observed the incremental benefit of LA MD or LAA MD for identifying stroke/TIA in three modeling steps. The initial model based on CHA2DS2-VASc score, iLAVmax, LAA dense SEC/Thrombus, LAAEV, LA and LAA GLS (χ^2^ = 14.35) was significantly improved by the addition of LAA MD (χ^2^ = 25.50, *P* < 0.01) and further improved by adding LA MD(χ^2^ = 32.41, *P* < 0 .01) (Fig. 4a). Similarly, the same initial model was also significantly improved by addition of LA MD (χ^2^ = 23.71, *P* < 0.01) and further improved by adding LA MD (χ^2^ = 32.41, *P* < 0.01) (Fig. 4b).

### The priorities of LA and LAA MD for risk stratification of stroke in patients with and without LA enlargement

**Fig. 5:**
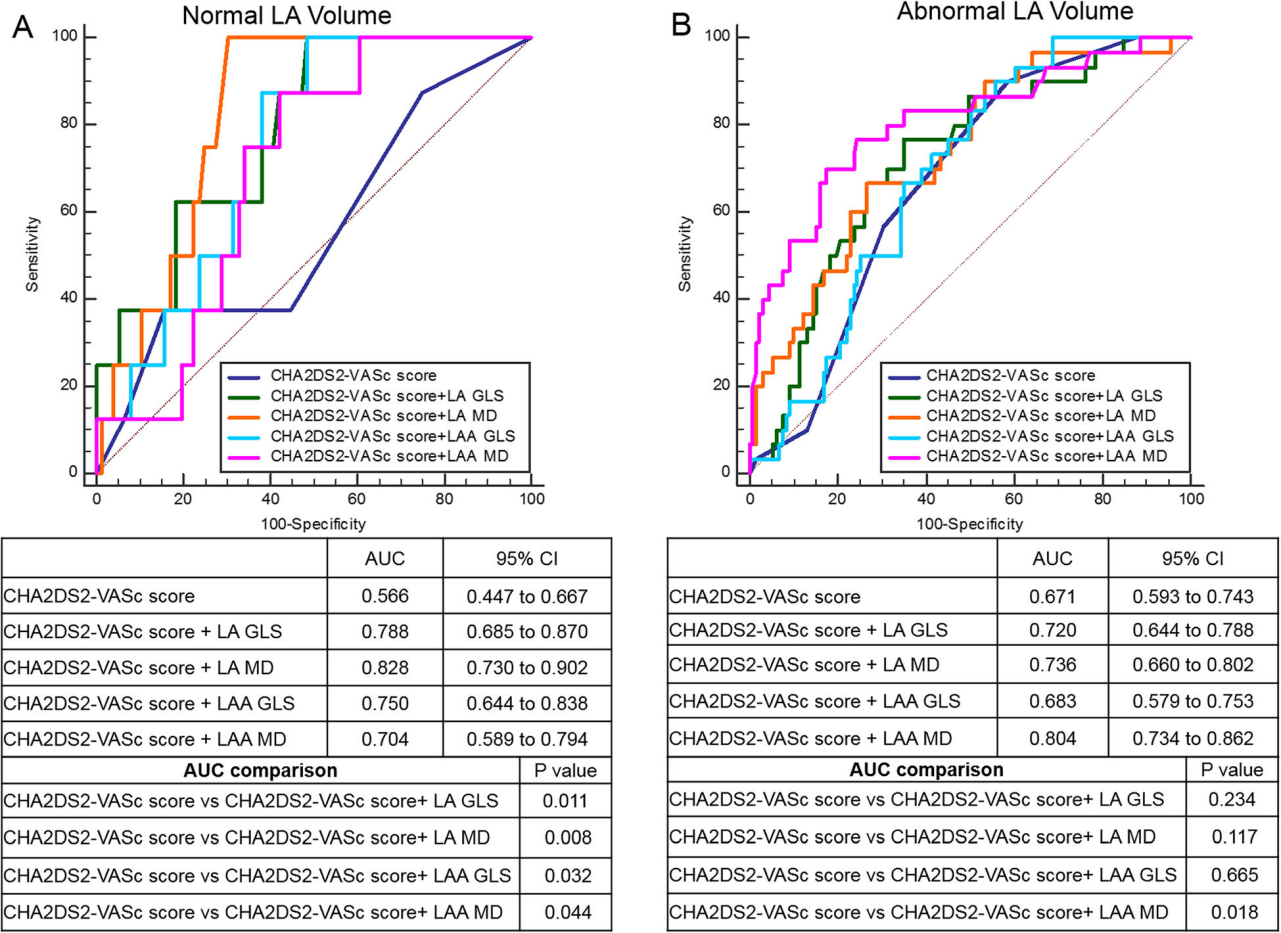
Results of receiver-operating characteristic curve analysis for identifying stroke in patients with normal LA volumes (iLAVmax < 34 mL/m^2^) (**a**) and patients with abnormal LA volumes (iLAVmax ≥34 mL/m^2^) (**b**). AUC, area under the curve; GLS, global longitudinal strain; i, indexed to body surface area; LA, left atrium; LAA, left atrial appendage; MD, mechanical dispersion; Vmax, maximal volume

We performed a subgroup analysis between patients with normal and abnormal LA size (Normal LA volume was defined as indexed LAV_max_ (iLAV_max_) < 34 mL/m^2^ [25]). Patients with LA enlargement were older, had higher prevalence of stroke and worse LA and LAA function than those with normal LA size (Supplementary data, Table 2). In the logistic regression analysis, the independent association of each variable with stroke/TIA was tested in six models (Table 3). In patients with LA enlargement, LAA MD was consistently identified as a significant contributor to previous stroke/TIA in every model but LA MD was not. Interestingly, in patients without LA enlargement, only LA MD was an independent discriminator for stroke. The incremental values of LA MD or LAA MD over CHA2DS2-VASc score, and either of LA GLS, LAEF, LAA EV, LAA GLS and LAA MD or LA MD were examined in both subgroups (Supplementary data, Figure 1). In patients with normal LA volumes, adding LA MD significantly improved all the five models, whereas, LAA MD provided no incremental value (Fig. 1a). However, only LAA MD had an additional diagnostic value for stroke in patients with LA enlargement (Fig. 1b). Furthermore, we assessed ROC analysis in both subgroups (Fig. 5). In patients with normal LA volumes, AUCs of CHA2DS2-VASc scores incorporated with both LA and LAA mechanics were significantly higher than that of CHA2DS2-VASc scores (Fig. 5a). On the other hand, in patients with LA enlargement, only AUC based on CHA2DS2-VASc score and LAA MD was significantly better compared with the CHA2DVASc score alone (0.804 vs 0.671, *P* = 0.018) (Fig. 5b).

**Table 3:** Univariable and multivariable binary logistic regression analysis for association with stroke in patients with normal (iLAVmax< 34 ml/m^2^) and abnormal (iLAVmax ≥34 ml/m^2^) LA volumes

**Norma LA volumes**	Univariable		Multivariable											
***N*** **= 81, stroke = 10**	OR (95% CI)	*P*	Model 1		Model 2		Model 3		Model 4		Model 5		Model 6	
			OR (95% CI)	*P*	OR (95% CI)	*P*	OR (95% CI)	*P*	OR (95% CI)	*P*	OR (95% CI)	*P*	OR (95% CI)	*P*
CHA2DS2-VASc score	1.26 (0.73–2.18)	0.40												
LA GLS (%)	0.88 (0.79–0.97)	0.01			0.97 (0.86–1.08)	0.56	0.88 (0.79–0.97)	0.01	0.86 (0.77–0.97)	< 0.01	0.87 (0.77–0.97)	< 0.01	0.88 (0.79–0.99)	0.04
LA MD (%)	1.51 (1.16–1.97)	< 0.01	1.51 (1.16–1.97)	< 0.01			1.51 (1.16–1.97)	< 0.01	1.85 (1.23–2.79)	< 0.01	1.55 (1.15–2.09)	< 0.01	1.66 (1.13–2.42)	< 0.01
LAEF(%)	0.94 (0.89–0.98)	0.01	0.97 (0.91–1.03)	0.28	0.97 (0.92–1.04)	0.40			0.94 (0.89–0.99)	0.02	0.95 (0.90–1.01)	0.11	0.95 (0.90–1.01)	0.09
LAAEV (cm/s)	1.96 (0.01–4.36)	0.70	0.87 (0.33–22.89)	0.21	1.00 (0.05–19.73)	0.89	1.97 (0.07–55.22)	0.24			1.00 (0.05–20.46)	0.98	0.47 (0.02–9.61)	0.63
LAA GLS(%)	0.80 (0.65–0.99)	0.05	0.90 (0.72–1.13)	0.36	0.92 (0.75–1.12)	0.39	0.80 (0.65–1.00)	0.05	0.80 (0.65–0.99)	0.05			0.85 (0.68–1.06)	0.15
LAA MD (%)	1.31 (1.06–1.61)	0.01	1.26 (0.99–1.61)	0.06	1.33 (0.99–1.77)	0.06	1.28 (1.02–1.60)	0.03	1.31 (1.06–1.61)	0.01	1.27 (1.01–1.59)	0.04		
**Abnormal LA volumes**	Univariable		Multivariable											
***N*** **= 127, stroke = 21**	OR (95% CI)	*P*	Model 1		Model 2		Model 3		Model 4		Model 5		Model 6	
			OR (95% CI)	*P*	OR (95% CI)	*P*	OR (95% CI)	*P*	OR (95% CI)	*P*	OR (95% CI)	*P*	OR (95% CI)	*P*
CHA2DS2-VASc score	1.47 (1.00–2.14)	0.05												
LA GLS (%)	0.93 (0.84–1.02)	0.10			0.99 (0.92–1.08)	0.33	0.93 (0.84–1.02)	0.10	0.89 (0.81–1.00)	0.05	0.93 (0.83–1.05)	0.26	0.90 (0.81–1.01)	0.07
LA MD (%)	1.18 (1.02–1.35)	0.03	1.18 (1.02–1.35)	0.03			1.18 (1.02–1.35)	0.01	1.21 (1.04–1.41)	0.01	1.17 (0.99–1.36)	0.05	1.16 (0.99–1.35)	0.06
LAEF (%)	0.99 (0.96–1.02)	0.41	1.01 (0.97–1.05)	0.90	0.98 (0.95–1.01)	0.54			0.99 (0.95–1.03)	0.56	0.99 (0.95–1.03)	0.65	0.99 (0.95–1.02)	0.42
LAAEV (cm/s)	0.02 (0.00–0.76)	0.04	0.15 (0.01–4.29)	0.34	0.10 (0.01–2.71)	0.16	0.03 (0.01–0.94)	0.05			0.06 (0.01–2.52)	0.14	0.05 (0.01–2.22)	0.12
LAA GLS (%)	0.85 (0.73–0.98)	0.02	0.84 (0.72–0.98)	0.02	0.91 (0.79–1.06)	0.06	0.84 (0.72–0.97)	0.02	0.85 (0.73–0.98)	0.02			0.91 (0.78–1.06)	0.22
LAA MD (%)	1.17 (1.05–1.31)	< 0.01	1.16 (1.04–1.30)	< 0.01	1.15 (1.03–1.29)	0.01	1.17 (1.05–1.30)	< 0.01	1.16 (1.04–1.30)	< 0.01	1.17 (1.05–1.31)	< 0.01		

Model 1 adjusted with CHA2DS2-VASc score and LA GLS

Model 2 adjusted with CHA2DS2-VASc score and LA MD

Model 3 adjusted with CHA2DS2-VASc score and LAEF

Model 4 adjusted with CHA2DS2-VASc score and LAAEV

Model 5 adjusted with CHA2DS2-VASc score and LAA GLS

Model 6 adjusted with CHA2DS2-VASc score and LAA MD

*AF* atrial fibrillation, *CI* Confidence intervals, *GLS* global longitudinal strain, *i* indexed to body surface area, *LA* left atrium, *LAA* left atrial appendage, *LAAEV* LAA emptying velocity, *LAEF* LA emptying fraction, *MD* mechanical dispersion, *OR* odds ratio, *Vmax* maximal volume

### Reproducibility

Intra-observer and inter-observer ICC were 0.984 (95% CI 0.955–0.994) and 0.981 (95% CI 0.945–0.993) for LA GLS, and 0.976 (95% CI 0.933–0.992) and 0.970 (95% CI 0.917–0.989) for LA MD; 0.967 (95% CI 0.909–0.988) and 0.955 (95% CI 0.879–0.984) for LAA GLS, and 0.964 (95% CI 0.903–0.987) and 0.945(95% CI 0.879–0.991) for LAA MD, respectively.

## Discussion

The major findings of this study were as follows: first of all, LA MD and LAA MD assessed by speckle-tracking echocardiography were independently associated with prior stroke/TIA in patients with nonvalvular AF. Secondly, both parameters provided incremental diagnostic values over clinical risk factors and conventional echocardiographic parameters. Interestingly, LA MD was more useful than LAA MD in patients with normal LA size, while LAA MD was superior to LA MD in patients with LA enlargement.

### LA and LAA mechanical dispersion as biomarkers for risk stratification of stroke/TIA

Currently, there is an ongoing exploration in the mechanisms of AF-related stroke, and LA/LAA remodeling, including structural and functional alterations, have been indicated as important underlying substrates for stroke [5, 6, 9–14, 28]. In addition to conventional LA parameters like LA dilation [5, 9], recent studies revealed that LA strain and mechanical dispersion could detect LA dysfunction in the absence of LA enlargement [29, 30] and had incremental diagnostic values for stroke over LA volume [16, 20, 31]. However, only one previous study [20] has linked LA MD to a history of stroke, which used tissue-tracking cardiac magnetic resonance (CMR) and was limited to patients during sinus rhythm. To the best of our knowledge, this is the first study to demonstrate the significant and independent contribution of LA MD to prior stroke/TIA using speckle-tracking echocardiography. LA MD > 11.47% accurately distinguished patients at higher risk of stroke. On the other hand, despite the potential role of LAA dysfunction as a marker of stroke, previous studies failed to demonstrate independent associations between LAA strain and embolic events [18, 21]. However, we can now detect LAA asynchrony using STE, and LAA MD has incremental value for embolism risk stratification in AF patients [21]. In the present study, the optimal LAA MD cutoff (> 12.97%) was completely consistent with our previous results [21] but in a larger sample size. We also investigated a comparison of both mechanical dispersions for the risk stratification of stroke, and identified that both variables were independent of and incremental to clinical, TTE and TEE parameters, including the other mechanical dispersion.

The mechanisms linking greater LA/LAA MD and stroke remain unclear. It is possible that inhomogenous atrial contraction may slow down the regional blood flow and lead to thrombogenesis in the LA/LAA. This is consistent with previous findings that higher atrial mechanical dispersion is associated with LAA thrombi or sludge [19, 32].

### Comparison of diagnostic values between LA and LAA MD

The present study demonstrated that usefulness of LA and LAA mechanics was different depending on LA size. In patients with normal LA volumes, stroke was thought to be mainly associated with LA dysfunction or ‘atrial cardiopathy’ because these patients are younger and had less traditional stroke risks than those with LA enlargement. Recent evidence supported that LA MD can detect LA dysfunction and asynchrony in the absence of LA enlargement [30]. Therefore, LA MD was superior to LAA MD in patients without LA enlargement. On the other hand, only LAA MD not LA MD had a consistently significant association with a history of stroke in patients with LA enlargement. In this subgroup, their LA function was significantly impaired, whereas, some non-stroke patients have preserved LAA function. That’s why LAA MD could provide incremental values in patients who already had more risk factor than those with normal LA volumes.

### Clinical implications

The findings in this study suggest that LA and LAA MD could be useful biomarkers to discriminate patients with strokes from controls, independent of and superior to CHA2DS2-VASc score. Therefore, assessment of LA and LAA dyssynchrony may help clinicians to identify patients at relatively higher risk for stroke. As LA/LAA remodeling are partially reversible [33, 34], therapies targeted at LA and LAA MD in addition to anticoagulation might provide benefits in patients with AF by improving LA/LAA mechanics.Future studies are warranted to test this hypothesis. In patients with LA enlargement, TEE screening can provide additional information for predicting stroke over TTE parameters. However, if the patients could not tolerate TEE or the TEE images were inadequate for strain analysis, LA MD may be an alternative to LAA MD, considering their comparable diagnostic values and greater technical difficulty in assessing LAA MD.

### Study limitations

The present study has several limitations and technical considerations. First of all, this is a single-centered, cross-sectional study composed of patients with moderate to low risk for stroke. Therefore, the selection bias may influence external validity of our results. Further prospective multicenter studies are needed to confirm our findings, including the cut-off values of LA and LAA MD. Secondly, the present study recorded stroke/TIA retrospectively from the index echocardiography. Ideally baseline strain measurements should be assessed before stroke onset, but this requires a larger sample size with longer follow-up. Third, although we used multiple views to generate LA and LAA MD, it is still possible that these measurements were underestimated due to missing regions that were not covered by these views. Fourth, although strain imaging is operator-dependent, intra- and interobserver reproducibility was excellent in our study. Finally, we analyzed LA and LAA strain using software for evaluating the LV because dedicated atrial strain packages are not available. In addition, vendor specificity of STE should also be considered.

## Conclusion

Higher LA and LAA mechanical dispersion assessed by speckle-tracking echocardiography are significantly and independently associated with a history of stroke/TIA in patients with AF and can provide incremental value for risk stratification of stroke over clinical and conventional echocardiographic parameters.

## Supplementary Information

**Additional file 1: Supplementary Table 1.** AUC for ROC analysis of clinical and echocardiographic variables. The variables that have the highest diagnostic performance for stroke in each group were highlighted. **Supplementary Table 2.** Baseline characteristics for subgroups with normal (iLAVmax < 34 mL/m^2^) and abnormal (iLAVmax≥34 mL/m^2^) LA volumes. **Figure 1.** The incremental value of LA MD or LAA MD for identifying stroke or transient ischaemic attack in six models in patients with normal LA volumes(iLAV_max_) < 34 mL/m^2^) (A) and abnormal LA volumes (iLAV_max_ ≥ 34 mL/m^2^) (B). AF, atrial fibrillation; GLS, global longitudinal strain; i, indexed to body surface area; LA, left atrium; LAA, left atrial appendage; LAAEV, LAA emptying velocity; LAEF, LA emptying fraction; MD, mechanical dispersion; V_max_, maximal volume

## Data Availability

The datasets used and/or analysed during the current study are available from the corresponding author on reasonable request.
