# Supplementary material for: Comparison of left atrial and left atrial appendage mechanics in the risk stratification of stroke in patients with atrial fibrillation
Source: Cardiovasc Ultrasound. 2021 Jan 9;19:7. doi: 10.1186/s12947-020-00232-z (PMC7797160; doi:10.1186/s12947-020-00232-z)
Supplement: Supplementary file 1 — Additional file 1: Supplementary Table 1. AUC for ROC analysis of clinical and echocardiographic variables. The variables that have the highest diagnostic performance for stroke in each group were highlighted. Supplementary Table 2. Baseline characteristics for subgroups with normal (iLAVmax < 34 mL/m2) and abnormal (iLAVmax≥34 mL/m2) LA volumes. Figure 1. The incremental value of LA MD or LAA MD for identifying stroke or transient ischaemic attack in six models in patients with normal LA volumes(iLAVmax) < 34 mL/m2) (A) and abnormal LA volumes (iLAVmax ≥ 34 mL/m2) (B). AF, atrial fibrillation; GLS, global longitudinal strain; i, indexed to body surface area; LA, left atrium; LAA, left atrial appendage; LAAEV, LAA emptying velocity; LAEF, LA emptying fraction; MD, mechanical dispersion; Vmax, maximal volume [file 12947_2020_232_MOESM1_ESM.docx]

SUPPLEMENTAL MATERIAL

Supplemental Tables

Table I AUC for ROC analysis of clinical and echocardiographic variables. The variables that have the highest diagnostic performance for stroke in each group were highlighted.

| **Variables** | **All patients** | | **Normal LA volume** | | **Abnormal LA volume** | |
| --- | --- | --- | --- | --- | --- | --- |
|  | **(N=208, stroke=31)** | | **(N=81, stroke=10)** | | **(N=127, stroke=21)** | |
|  | AUC | 95%CI | AUC | 95%CI | AUC | 95%CI |
| **Clinical parameters** |  |  |  |  |  |  |
| CHA_2_DS_2_-VASc score | 0.666 | 0.604-0.725 | 0.566 | 0.447- 0.667 | 0.671 | 0.593-0.743 |
| **LA parameters** |  |  |  |  |  |  |
| iLAVmax (ml/m^2^) | 0.633 | 0.571-0.692 | 0.603 | 0.550-0.765 | 0.661 | 0.602-0.771 |
| iLAVmin (ml/m^2^) | 0.631 | 0.561-0.697 | 0.585 | 0.470-0.693 | 0.676 | 0.637-0.801 |
| LAEF,% | 0.647 | 0.577-0.712 | 0.723 | 0.653-0.826 | 0.561 | 0.469-0.651 |
| LA GLS,% | 0.711 | 0.652-0.764 | 0.726 | 0.600-0.812 | 0.685 | 0.610-0.754 |
| LA MD,% | 0.724 | 0.666-0.777 | 0.780 | 0.682-0.859 | 0.694 | 0.619-0.762 |
| **LAA parameters** |  |  |  |  |  |  |
| LAAEV | 0.623 | 0.551-0.692 | 0.559 | 0.449-0.664 | 0.666 | 0.574-0.749 |
| LAAEF | 0.611 | 0.538-0.680 | 0.636 | 0.584-0.718 | 0.597 | 0.504-0.686 |
| LAA GLS | 0.699 | 0.631-0.765 | 0.756 | 0.652-0.843 | 0.667 | 0.579-0.753 |
| LAA MD | 0.771 | 0.714-0.822 | 0.705 | 0.597-0.799 | 0.788 | 0.718-0.848 |

AUC, area under the curve ;CI, Confidence intervals; GLS, global longitudinal strain; i, indexed - body surface area; LA, left atrium; LAA, left atrial appendage; LAAEF, LAA emptying fraction; LAAEV, LAA emptying velocity; LAEF, LA emptying fraction; MD, mechanical dispersion; ROC, Receiver operating characteristic ; Vmax, maximal volume; Vmin, minimal volume.

Table II Baseline characteristics for subgroups with normal (iLAVmax < 34 mL/m^2^) and abnormal (iLAVmax≥34 mL/m^2^) LA volumes

|  | **Normal LA volume**  **(N=81)** | **Abnormal LA volume**  **(N=127)** | | | P value | |  |
| --- | --- | --- | --- | --- | --- | --- | --- |
| **Clinical characteristics** |  |  | | |  | |  |
| gender,male | 54(66.7) | | 79(62.2) | | 0.51 | |  |
| Age, years | 60.06±11.75 | | 65.82±8.73 | | <0.01 | |  |
| Body mass index, kg/m^2^ | 23.93±3.12 | | 24.66±3.22 | | 0.11 | |  |
| Heart failure | 4(4.9) | | 29(22.8) | | <0.01 | |  |
| Coronary artery disease | 15(18.5) | | 22(17.3) | | 0.83 | |  |
| Hypertension | 37(45.7) | | 74(58.3) | | 0.08 | |  |
| Diabetes | 20(24.7) | | 25(19.7) | | 0.39 | |  |
| Hyperlipoproteinemia | 26(32.1) | | 32(25.2) | | 0.28 | |  |
| Persistent AF | 31(38.3) | | 89(70.1) | | <0.01 | |  |
| Prior stroke/TIA | 10(12.3) | | 21(16.5) | | 0.41 | |  |
| Anticoagulation | 52(64.2) | | 93(73.2) | | 0.17 | |  |
| CHA2DS2-VASc score | 1.51±1.18 | | 2.02±1.27 | | <0.01 | |  |
| **Conventional echocardiographic parameters** | | | | | | |  |
| iLVESV,mL/m2 | 21.28±6.49 | | 26.04±10.48 | | <0.01 | |  |
| iLVEDV,mL/m^2^ | 65.09±13.82 | | 67.96±14.95 | | 0.17 | |  |
| LVEF,% | 67.18±7.13 | | 62.39±8.38 | | <0.01 | |  |
| iLAAPd ,mm/m^2^ | 20.71±2.76 | | 24.09±3.82 | | <0.01 | |  |
| iLAVmin, mL/m^2^ | 15.06±4.70 | | 36.25±14.30 | | <0.01 | |  |
| iLAVmax, mL/m^2^ | 26.24±5.15 | | 51.83±14.35 | | <0.01 | |  |
| LAEF,% | 42.74±13.86 | | 30.23±15.41 | | <0.01 | |  |
| LAAEV,m/s | 0.67±0.23 | | 0.44±0.20 | | <0.01 |  |  |
| LAAFV,m/s | 0.60±0.17 | 0.48±0.21 | | | <0.01 | |  |
| LAA dense SEC/thrombus | 2(2.7) | 34(28.1) | | | <0.01 | |  |
| ilAAVmin,mL/m^2^ | 1.07±0.81 | 2.41±1.73 | | | <0.01 | |  |
| iLAAVmax,mL/m^2^ | 3.15±1.81 | 4.92±2.49 | | | <0.01 | |  |
| LAAEF,% | 65.03±18.15 | 52.60±19.40 | | | <0.01 | |  |
| **Strain echocardiographic parameters** | | | | | | | |
| LA GLS,% | 24.17±10.04 | 14.14±6.97 | | <0.01 | | |  |
| LA MD,% | 7.11±3.53 | 10.08±3.44 | | <0.01 | | |  |
| LAA GLS,% | 14.88±6.91 | 10.53±4.35 | | <0.01 | | |  |
| LAA MD,% | 10.54±4.55 | 12.12±4.83 | | 0.03 | | |  |

Data are expressed as mean±SD or N (%).

AF ,atrial fibrillation; EDV end-diastolic volume; ESV end-systolic volume; GLS, global longitudinal strain; i, indexed to body surface area; LA, left atrium; LAA, left atrial appendage; LAAEF, LAA emptying fraction; LAAEV, LAA emptying velocity; LAAFV, LAA filling velocity; LAAPd, LA anteroposterior diameter; LAEF,LA emptying fraction; LV, left ventricle; LVEF, left ventricular ejection fraction; MD,mechanical dispersion; SEC, spontaneous echo contrast; TIA, transient ischaemic attack ;Vmax,maximal volume; Vmin,minimal volume.

Supplemental Figures and Figure Legends


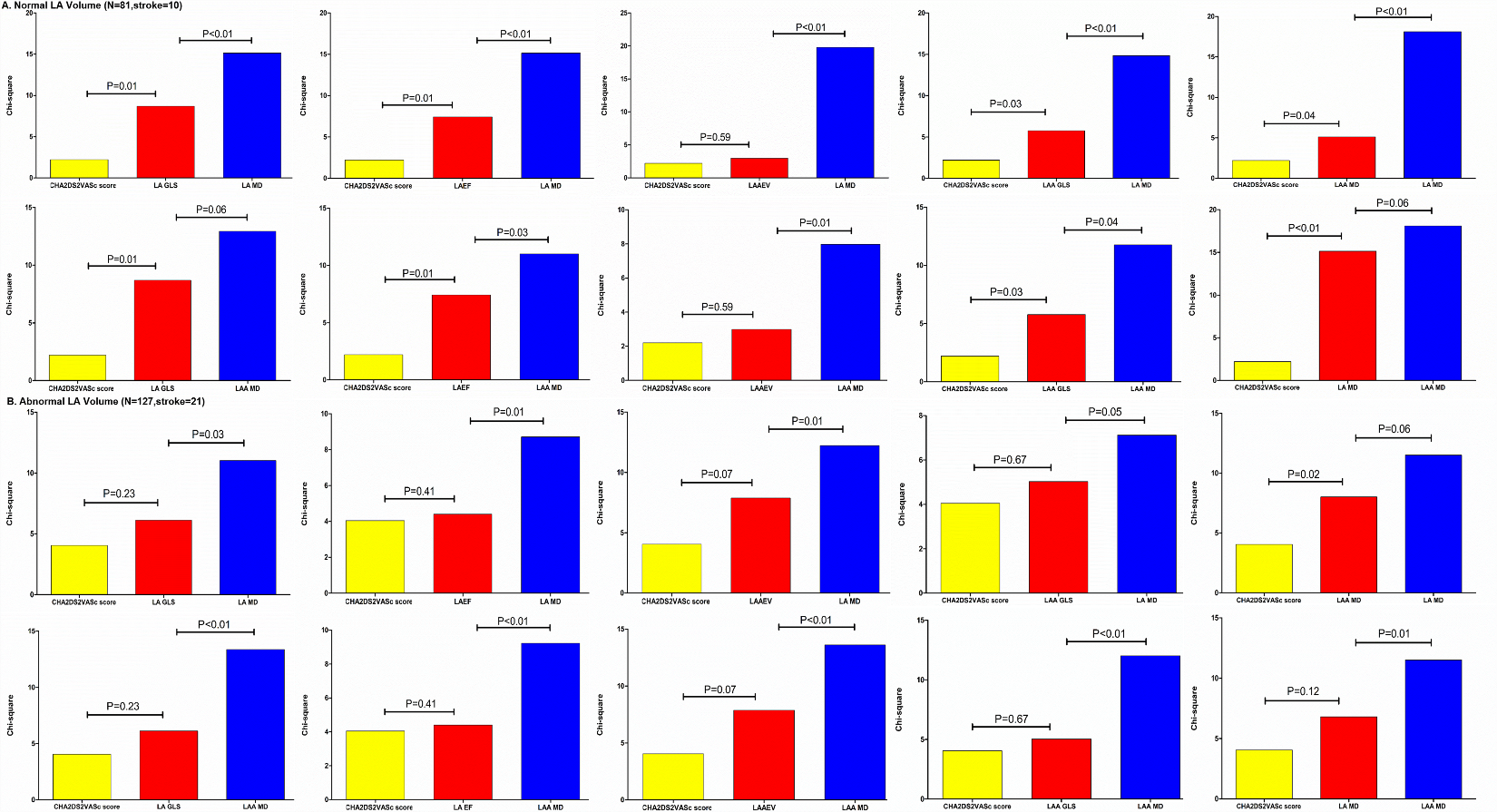


**Figure I** The incremental value of LA MD or LAA MD for identifying stroke or transient ischaemic attack in six models in patients with normal LA volumes(iLAV_max_) <34 mL/m^2^) (A) and abnormal LA volumes (iLAV_max_ ≥34mL/m^2^) (B). AF, atrial fibrillation; GLS, global longitudinal strain; i, indexed to body surface area; LA, left atrium; LAA, left atrial appendage; LAAEV, LAA emptying velocity; LAEF, LA emptying fraction; MD, mechanical dispersion; V_max_, maximal volume.
